# Knowledge and attitudes towards abortion among young adults in Pahang, Malaysia: A descriptive cross-sectional study

**DOI:** 10.51866/oa.871

**Published:** 2026-03-30

**Authors:** Nurhazirah Zainul Azlan, Nur Anisah Ashila Safari, Noratikah Othman, Muhammad Lokman Md. Isa

**Affiliations:** 1 Department of Basic Medical Sciences for Nursing, Kulliyyah of Nursing, International Islamic University Malaysia (IIUM), Kuantan, Pahang, Malaysia.; 2 Institute of Planetary Survival for Sustainable Well-being (PLANETIIUM), International Islamic University Malaysia (IIUM), Kuantan, Pahang, Malaysia.; 3 Operating theatre, Subang Jaya Medical Centre, SJP Medical Centres Sdn.Bhd (Formerly known as RSD Hospitals Sdn. Bhd.), c/o Subang Jaya Medical Centre, Subang Jaya, Selangor, Malaysia.

**Keywords:** Knowledge, Attitude, Abortion, Young adult, Malaysia

## Abstract

**Introduction:**

Abortion remains a sensitive issue in Malaysia due to sociocultural and religious factors. Young adults with limited reproductive health awareness and support are vulnerable to unintended pregnancies and unsafe abortion. Therefore, this study aimed to assess young adults’ knowledge and attitudes towards unsafe abortion and their association with sociodemographic characteristics in Kuantan, Pahang.

**Methods:**

A descriptive cross-sectional study using stratified random sampling was conducted among 230 young adults aged 15–24 years in Kuantan, Pahang. Data were collected through questionnaires and analysed using the Statistical Package for the Social Sciences version 28.0.

**Results:**

Most respondents were aware that unsafe abortions are performed by unskilled individuals and within unsafe environments and include swallowing harmful chemical, inserting foreign objects/substances into the uterus and ingesting caustic substances. The respondents agreed that government-provided abortion services can increase inappropriate sexual behaviours (63.0%) and that legal restrictions can lead to unsafe abortions (61.7%). Many respondents believed that abortion should be allowed for cases including foetal abnormalities (58.4%) and rape (40.9%) but not for Down syndrome (40.4%) and unmarried pregnant adolescents (45.2%). Additionally, age and educational level as well as religion and educational level were associated with knowledge and attitudes towards unsafe abortion, respectively (P<0.05).

**Conclusion:**

Enhanced awareness programmes are needed, as many young adults demonstrated knowledge towards unsafe abortion yet mixed attitudes influenced by sociocultural and religious beliefs. Targeted interventions are crucial to address gaps in age, education and religious beliefs for ensuring equitable access to reproductive health services among young adults.

## Introduction

Abortion is a common gynaecological procedure among women with the purpose of ending their pregnancy.^1^ According to the World Health Organization (WHO), abortion is safe if the medical procedure is conducted by trained personnel within a suitable duration of gestation.^2^ There are several types of abortions, such as spontaneous abortion or miscarriage, induced abortion (an interruption of pregnancy by medical or surgical intervention), incomplete abortion and foetal death. In 2024, the WHO reported that approximately 45% of all abortions were unsafe, making unsafe abortion a significant preventable cause of maternal deaths and morbidities. This subsequently results in physical and mental health challenges alongside social and financial strains on women, communities and healthcare systems.^3^

Unsafe abortion is defined as a pregnancy termination procedure conducted by individuals without the required knowledge or skills, in settings that fail to meet basic medical standards or both.^3^ There are several methods to terminate pregnancy through unsafe abortion, including drinking toxic fluids (e.g. turpentine or bleach), inflicting direct injury to the vagina and cervix by inserting herbs, putting a foreign body (e.g. chicken bones) into the uterus or using unsuitable medications. Unsafe abortion can also occur through dilatations and curettages performed in an unsanitary environment, resulting in uterine perforations and infections. Additionally, some unsafe abortions are performed by jumping from the top of stairs or a roof, causing blunt trauma to the pregnant woman’s abdomen.^4^

According to the global estimates by the WHO, approximately 73 million induced abortions occur annually. From this figure, the prevalence of unsafe abortion accounts for around 45%, with 97% of cases occurring in developing countries. Both South and Central Asia have the highest rate of unsafe abortions in Asia, whereas Latin America and Africa have the highest rate among Western countries.^5^ Furthermore, 4.7%–13.2% of annual maternal deaths are caused by unsafe abortion.^6^ In developed regions, approximately 30 women die per 100,000 unsafe abortions. This figure increases to 220 deaths per 100,000 unsafe abortions in developing regions and rises further to 520 deaths per 100,000 unsafe abortions in sub-Saharan Africa.^7^ The United Nations Population Fund also postulated unsafe abortion as among the leading causes of maternal deaths worldwide,^8^ with 7 million women being hospitalised every year due to major complications from unsafe abortion.

The age range of 15–24 years was selected for this study based on definitions from previous studies that identify youth or young people as individuals within this age group.^9-11^ This age range represents a transitional period characterised by the initiation of sexual activity, the development of personal identity and the tendency towards increased risk-taking behaviours, such as engaging in unprotected sex. In 2019, 55% of unintended pregnancies involving girls aged 15 years and above resulted in abortions, many of which were unsafe in low- and middle-income countries.^12^ Previous research has shown that young people within this age range often begin engaging in sexual activities due to being influenced by peer acceptance of premarital sex despite adult disapproval. They also have limited knowledge of contraceptive methods, and access to contraceptives is often hindered by factors such as cost, shame, stigma and judgemental attitudes from healthcare providers.^9^ Therefore, targeting this population is considered crucial for improving awareness and preventing adverse abortion-related outcomes.

However, in Malaysia, the lack of available data on abortion creates a significant gap in public knowledge regarding the issue. Previous research has revealed that Malaysia has no documented data on complications related to induced abortion, which can serve as an indicator of whether safe or unsafe abortions are more commonly practised in this country. Moreover, the actual number of cases from surviving mothers living with morbidity or negative health outcomes due to unsafe abortion is also undocumented.^13^ This is in addition to the lack of studies regarding the knowledge and attitudes towards unsafe abortion among local populations in Malaysia. Therefore, the present study aimed to assess the levels of knowledge and attitudes towards unsafe abortion and determine their interplay with sociodemographic characteristics among young adults aged 15-24 years in Kuantan, Pahang, Malaysia.

## Methods

This quantitative study adopted a descriptive cross-sectional research design involving stratified random sampling. The sample size was calculated using the Raosoft, Inc. software, which applied the single-proportion formula with the 557,800 population of Kuantan, Pahang. The parameters used were 5% margin of error, 85% confidence level and 50% response distribution. This approach ensured a statistically adequate and representative research sample. The minimum sample size for this study was 229 respondents, with a 10% dropout rate. Respondents were selected among 230 young adults aged 15-24 years in Kuantan, Pahang. Male and female individuals aged 15-24 years were included in the study, while foreign young adults who were not residing in Kuantan, Pahang, were excluded.

The questionnaire used in this study was adapted from the previous studies.^14-18^ Permission to use the established questionnaire was obtained from the respective authors. The questionnaire utilised in the present study comprised three parts: Part A gathered sociodemographic data; Part B contained questions regarding the knowledge of unsafe abortion, including the causes, methods and complications of unsafe abortion; and Part C explored the attitudes towards unsafe abortion. The scoring approach was adapted from previously published studies. The level of knowledge regarding unsafe abortion was assessed using Yes/No questions, with each correct response was scored 1 and incorrect response was scored 0. The score for attitude level was given using three- and four-point Likert scale, with higher score indicating a more appropriate attitude towards unsafe abortion.

The English version of the questionnaire was distributed to respondents using both online and offline methods. The online method involved creating the questionnaire using Google Forms and sharing it via social media platforms (e.g. WhatsApp and Facebook) to reach young adults residing in Kuantan, Pahang. The offline method involved distributing the questionnaire at public recreational areas commonly frequented by young adults. Respondents were allowed to seek clarification on any items they found unclear during data collection to eliminate potential comprehension issues due to variations in English proficiency. Such measure was implemented to enhance the accuracy and reliability of the responses.

This study obtained ethical approvals from the Kulliyyah of Nursing Postgraduate Research Committee and the IIUM Research Committee (IREC), with approval number IREC 2021-KON/UG13. Informed consent was first obtained from respondents who volunteered to participate in the study; they were free to withdraw from the study or decline to answer the questionnaire at any time. Data were analysed using the IBM Statistical Package for the Social Sciences version 28.0. A descriptive statistical test was used to measure the frequency and percentage of the variables, with P-values less than 0.05 considered statistically significant. The scoring for both level of knowledge and attitude was summed to obtain the total score and were then converted into percentage scores (0–100) and presented as median and interquartile range (IQR) due to the non-normal distribution of the data. The association between the sociodemographic characteristics and the levels of knowledge and attitudes towards unsafe abortion was analysed using the Kruskal–Wallis test.

## Results

### Sociodemographic data

Table 1 shows that the majority of the respondents were Malays (97.0%), Muslims (97.0%), single (99.6%), women (89.6%), students (88.3%), degree holders (79.1%), aged 23–24 years (47.0%), lived in urban areas (66.5%) and belonged to the B40 family income group (62.2%).

**Table 1:** Sociodemographic characteristics of the respondents (N=230).

Variable		n	%
Age	15–16 years	5	2.2
	17–18 years	9	3.9
	19–20 years	48	20.9
	21–22 years	60	26.1
	23–24 years	108	47.0
Sex	Male	24	10.4
	Female	206	89.6
Occupation	Student	203	88.3
	Unemployed	6	2.6
	Self-employed	4	1.7
	Private	14	6.1
	Government	3	1.3
Monthly household income	B40 (<RM 4850)	143	62.2
	M40 (RM 4851-10,970)	66	28.7
	T20 (>RM 10,971)	21	9.1
Ethnicity	Malay	223	97.0
	Chinese	5	2.2
	Indian	2	0.9
Religion	Islam	223	97.0
	Buddhism	5	2.2
	Hinduism	2	0.9
Residency	Urban	153	66.5
	Rural	77	33.5
Marital status	Single	229	99.6
	Married	1	0.4
Educational level	Primary education	3	1.3
	Secondary education	42	18.3
	Degree	182	79.1
	PhD or master’s degree	3	1.3

### Knowledge regarding unsafe abortion among young adults

Table 2 demonstrates that most respondents agreed that abortion should be a woman’s personal decision (66.1%) and that unsafe abortion is mainly performed by the pregnant woman herself (87.8%), untrained personnel at the hospital (77.4%), trained personnel at their home (66.5%) and older adult women in the community (75.2%). The respondents also agreed that unsafe abortion occurs when a pregnancy is terminated by individuals lacking the necessary skills (88.3%) or by untrained persons using dangerous methods such as inserting foreign bodies or using traditional concoctions (98.3%), when it is performed in an environment that does not conform to minimal medical standards (91.7%) and when it involves swallowing harmful chemical (93.0%), inserting foreign objects/substances through the vagina into the uterus (95.2%) or ingesting caustic substances (90.9%).

**Table 2:** Knowledge regarding unsafe abortion among young adults (N=230).

Variable	n (%)	
	Yes	No
Heard about abortion	227 (98.7)	3 (1.3)
Abortion should be a woman’s personal decision.	152 (66.1)	78 (33.9)
Unsafe abortion is done by self.	202 (87.8)	28 (12.2)
Unsafe abortion is done by a doctor at the hospital.	47 (20.4)	183 (79.6)
Unsafe abortion is done by untrained personnel at the hospital.	178 (77.4)	52 (22.6)
Unsafe abortion is done by trained personnel at my or their home.	153 (66.5)	77 (33.5)
Unsafe abortion is done by an older adult woman in the community.	173 (75.2)	57 (24.8)
Unsafe abortion is a simple, risk-free procedure.	85 (37.0)	145 (63.0)
Unsafe abortion can result in death.	227 (98.7)	3 (1.3)
Unsafe abortion can lead to haemorrhage.	221 (96.1)	9 (3.9)
Unsafe abortion can lead to subfertility.	220 (95.7)	10 (4.3)
Unsafe abortion can lead to uterine infections.	224 (97.4)	6 (2.6)
Abortion is not legally allowed in Malaysia except to prevent the death of the mother.	209 (90.9)	21 (9.1)
There are illegal places where abortions are carried out in the country.	208 (90.4)	22 (9.6)
Unsafe abortion occurs when a pregnancy is terminated by persons lacking the necessary skills.	203 (88.3)	27 (11.7)
Unsafe abortion occurs in an environment that does not conform to minimal medical standards.	211 (91.7)	19 (8.3)
Abortion is unsafe when it involves the ingestion of caustic substances.	209 (90.9)	21 (9.1)
Abortion is unsafe when untrained personnel use dangerous methods, such as inserting foreign bodies or using traditional concoctions.	226 (98.3)	4 (1.7)
Unintended pregnancy is a major driving force for unsafe abortion.	219 (95.2)	11 (4.8)
Any woman with an unwanted pregnancy who cannot access safe abortion is at risk for unsafe abortion.	220 (95.7)	10 (4.3)
Unsafe abortion may involve swallowing harmful chemicals.	214 (93.0)	16 (7.0)
Unsafe abortion involves the insertion of foreign objects/substances through the vagina into the uterus.	219 (95.2)	11 (4.8)
Several complications from unsafe abortion contribute to maternal sickness and sometimes to long-term disability and maternal death.	219 (95.2)	11 (4.8)
Infertility or inability to bear children may be a consequence of past unsafe abortion.	213 (92.6)	17 (7.4)
There is a link between unsafe abortion and a lack of social support system for pregnant adolescents.	220 (95.7)	10 (4.3)
Prevention of unintended pregnancy through the use of emergency contraception can reduce the burden of unsafe abortion.	212 (92.2)	18 (7.8)
Abortion is allowed if the pregnancy endangers the woman’s life.	223 (97.0)	7 (3.0)
Abortion is allowed if the child might be born deformed.	169 (73.5)	61 (26.5)
Abortion is allowed if the pregnancy resulted from rape.	94 (40.9)	136 (59.1)
Abortion is allowed if the family cannot afford to support the child.	51 (22.2)	179 (77.8)
Abortion is allowed if the woman is not married.	34 (14.8)	196 (85.2)
Abortion is allowed if the young girl or woman wants to continue her studies.	39 (17.0)	191 (83.0)
Unsafe abortion can lead to death.	225 (97.8)	5 (2.2)
Unsafe abortion can cause infertility.	217 (94.3)	13 (5.7)
Unsafe abortion can lead to womb damage or problems.	228 (99.1)	2 (0.9)
Unsafe abortion causes severe bleeding.	226 (98.3)	4 (1.7)
Unsafe abortion causes infection.	223 (97.0)	7 (3.0)
Unsafe abortion results in negative mental or psychological impacts.	221 (96.1)	9 (3.9)

A descriptive analysis was conducted.

The majority of the respondents also agreed that unsafe abortion is mainly driven by unintended pregnancy (95.2%) and limited to no access to safe abortion by women with an unwanted pregnancy (95.7%). The respondents also advocated the link between unsafe abortion and a lack of social support system for pregnant adolescents (95.7%) and that preventing unintended pregnancy through the use of emergency contraception can reduce the burden of unsafe abortion (92.2%).

Moreover, many respondents believed that abortion can be permitted if the pregnancy endangers the woman’s life (97.0%) or if the child might be born deformed (73.5%). Nevertheless, the respondents believed that abortion should not be allowed if the pregnancy is caused by rape (59.1%), if the family cannot afford to support the child (77.8%), if the woman is unmarried (85.2%) and if the pregnant young girl or woman wants to continue her studies (83.0%).

### Attitudes towards unsafe abortion among young adults

Table 3 illustrates the respondents’ view that unsafe abortion is a problem (97.4%), a sin (82.6%), common among students (53.5%) and considered normal nowadays (50.9%). The respondents agreed that a pregnant woman should have the right whether to opt for abortion (46.1%). Most respondents also believed that induced abortion should be restricted only to save a mother’s life (73.0%) and that it should not be allowed under other circumstances (37.0%), as making abortion services available through government health facilities can increase inappropriate sexual behaviours among people (63.0%).

**Table 3:** Attitudes towards unsafe abortion among young adults (N=230).

Attitude towards unsafe abortion	n (%)		
	Common	Not common	I do not know
How common is unsafe abortion among students?	123 (53.5)	34 (14.8)	73 (31.7)
	**Agree**	**Neutral**	**Disagree**
Is unsafe abortion a problem?	224 (97.4)	5 (2.2)	1 (0.4)
Would abort if pregnant	33 (14.3)	50 (21.7)	147 (63.9)
Abortion is a sin.	190 (82.6)	20 (8.7)	20 (8.7)
Considered normal nowadays	117 (50.9)	46 (20.0)	67 (29.1)
Do you think that abortion is lawful in Malaysia?	81 (35.2)	60 (26.1)	89 (38.7)
A pregnant woman should have the right to decide whether she wants abortion.	106 (46.1)	86 (37.4)	38 (16.5)
Induced abortion should be restricted to save a mother’s life.	168 (73.0)	47 (20.4)	15 (6.5)
Induced abortion should not be allowed under any circumstances.	85 (37.0)	84 (36.5)	61 (26.5)
Offering abortion services through government healthcare facilities can increase inappropriate sexual behaviours among people.	145 (63.0)	58 (25.2)	27 (11.7)
Induced abortion should be freely available in the country on demand to anyone who needs it.	54 (23.5)	84 (36.5)	92 (40.0)
Induced abortion should be freely available in the country on demand only to married couples.	63 (27.4)	86 (37.4)	81 (35.2)
Abortion can be considered a more convenient form of family planning than contraceptives.	46 (20.0)	60 (26.1)	124 (53.9)
Legal restrictions drive people to unsafe abortions and fall into trouble.	142 (61.7)	66 (28.7)	22 (9.6)
Abortion is an accepted way to solve unwanted pregnancy.	54 (23.5)	47 (20.4)	129 (56.1)
A mother should feel obligated to bear a child she has conceived.	151 (65.7)	59 (25.7)	20 (8.7)
Every conceived child has the right to be born.	188 (81.7)	31 (13.5)	11 (4.8)
Abortion is permissible if pregnancy is a threat the to mother’s life.	202 (87.8)	20 (8.7)	8 (3.5)
Abortion should occur if the foetus has congenital anomalies or genetic diseases.	134 (58.3)	71 (30.9)	25 (10.9)
Abortion must be considered if the foetus has Down syndrome.	60 (26.1)	77 (33.5)	93 (40.4)
Abortion must be considered murder.	99 (43.0)	81 (35.2)	50 (21.7)
People should not look inferior to women who choose to have an abortion.	130 (56.5)	76 (33.0)	24 (10.4)
Abortion should be an option available to unmarried pregnant adolescents.	48 (20.9)	78 (33.9)	104 (45.2)
Abortion should be available to women who conceived from rape.	94 (40.9)	84 (36.5)	52 (22.6)
The foetus must be considered human from the moment of conception.	125 (54.3)	73 (31.7)	32 (13.9)
Abortion is permissible before the soul is breathed into the foetus and not after it.	122 (53.0)	80 (34.8)	28 (12.2)
Discussion with patients about abortion is a social flaw.	109 (47.4)	87 (37.8)	34 (14.8)
Laws and legislation will support abortion in the future because of changes in the culture of the society.	95 (41.3)	88 (38.3)	47 (20.4)
Abortion must be prevented because I am not against abortion.	92 (40.0)	59 (25.7)	79 (34.3)
Abortion must be prevented because it is against my religion.	178 (77.4)	21 (9.1)	31 (13.5)
Abortion must be prevented because it is against tradition and norms.	144 (62.6)	41 (17.8)	45 (19.6)
Abortion must be prevented because it is an act of murder.	153 (66.5)	42 (18.3)	35 (15.2)

Variable	n (%)			
	Strongly disagree	Disagree	Agree	Strongly agree
Women seeking abortion are considered promiscuous.	33 (14.3)	53 (23.0)	120 (52.2)	24 (10.4)
Abortion is committing a sin.	28 (12.2)	33 (14.3)	106 (46.1)	63 (27.4)
Community belief on abortion is acceptable if the gestational age is below 3 months.	32 (13.9)	40 (17.4)	131 (57.0)	27 (11.7)
Women should have access to safe abortion services.	27 (11.7)	22 (9.6)	130 (56.5)	51 (22.2)
A woman can die from an abortion done in unsafe conditions or by untrained providers.	22 (9.6)	4 (1.7)	88 (38.3)	116 (50.4)
It is not acceptable to talk about any abortion-related issues.	59 (25.7)	89 (38.7)	56 (24.3)	26 (11.3)

While the majority of the respondents believed that every conceived child has the right to be born (81.7%), their opinion towards abortion varied significantly. Some were of the opinion that a foetus is considered a human being from the moment of conception (54.3%). The respondents subsequently rejected the idea of abortion (63.9%), as it was deemed an act of murder (66.5%), which was against both religion (77.4%) and traditions and norms (62.6%). Conversely, there were respondents who thought that abortion should be permissible before a soul is breathed into the foetus and not after it (53.0%). The respondents believed that abortion should be made available to those in need (40.0%), particularly if the pregnancy imposes a threat to the mother’s life (87.8%), if the foetus has potential congenital anomalies or genetic diseases (58.3%) or if the woman conceives from rape (40.9%). However, the respondents disagreed that abortion can be considered a more convenient method for family planning than contraceptives (53.9%), an acceptable way to solve cases of unwanted pregnancy (56.1%) or high-risk pregnancy such as a foetus with Down syndrome (40.4%) and an option available to unmarried pregnant adolescents (45.2%).

### Association between the sociodemographic characteristics and level of knowledge towards unsafe abortion

Table 4 demonstrates the association of age (P=0.0001) and level of education (P=0.005) with the level of knowledge towards unsafe abortion.

**Table 4:** Association of the sociodemographic characteristics with the level of knowledge towards unsafe abortion (N=230).

Variable		n (%)	Knowledge Score - Median (IQR)	P-value
Age	15–16 years	5 (2.2)	66 (10.0)	
	17–18 years	9 (3.9)	71 (4.50)	
	19–20 years	48 (20.9)	71 (2.75)	0.0001^*^
	21–22 years	60 (26.1)	72 (4.00)	
	23–24 years	108 (47.0)	73 (3.00)	
Sex	Male	24 (10.4)	71 (3.75)	0.351
	Female	206 (89.6)	72 (2.25)	
Ethnicity	Malay	223 (97.0)	72 (3.00)	
	Chinese	5 (2.2)	72 (6.50)	0.881
	Indian	2 (0.9)	71 (-)	
Occupation	Unemployed	6 (2.6)	71.5 (3.75)	
	Self-employed	4 (1.7)	65.5 (6.25)	
	Student	203 (88.3)	72 (4.00)	0.119
	Private	14 (6.1)	72 (2.75)	
	Government	3 (1.3)	74 (-)	
Religion	Islam	223 (97.0)	72 (3.00)	
	Buddhism	5 (2.2)	72 (6.50)	0.887
	Hinduism	2 (0.9)	71 (-)	
Monthly household income	B40 (<RM 4850)	143 (62.2)	72 (3.00)	
	M40 (RM 4851-10,971)	66 (28.7)	72 (2.00)	0.216
	T20 (>RM 10,971)	21 (9.1)	73 (2.00)	
Marital status	Single	229 (99.6)	72 (3.00)	0.145
	Married	1 (0.4)	-	
Educational level	Primary education	3 (1.3)	71 (-)	
	Secondary education	42 (18.3)	71 (5.00)	
	Degree	182 (79.1)	72 (3.00)	0.005^*^
	PhD or master’s degree	3 (1.3)	72 (-)	

* Kruskal–Wallis test, P<0.05

### Association of the sociodemographic characteristics with the level of attitudes towards unsafe abortion

Table 5 shows the association of religion (P=0.038) and level of education (P=0.042) with the level of attitudes towards unsafe abortion.

**Table 5:** Association of the sociodemographic characteristics with the level of attitudes towards unsafe abortion.

Variable		n (%)	Attitude score - Median (IQR)	P-value
Age	15-16 years	5 (2.2)	80 (12.50)	
	17-18 years	9 (3.9)	77 (5.50)	
	19-20 years	48 (20.9)	73 (26.75)	0.256
	21-22 years	60 (26.1)	75 (8.00)	
	23-24 years	108 (47.0)	76.5 (9.00)	
Sex	Male	24 (10.4)	77.5 (11.50)	0.351
	Female	206 (89.6)	76 (11.00)	
Ethnicity	Malay	223 (97.0)	76 (10.00)	
	Chinese	5 (2.2)	81 (5.00)	0.070
	Indian	2 (0.9)	69 (-)	
Occupation	Unemployed	6 (2.6)	72 (12.50)	
	Self-employed	4 (1.7)	83 (9.75)	
	Student	203 (88.3)	76 (11.00)	0.074
	Private	14 (6.1)	76 (7.75)	
	Government	3 (1.3)	77 (-)	
Religion	Islam	223 (97.0)	76 (10.00)	
	Buddhism	5 (2.2)	81 (5.00)	0.038^*^
	Hinduism	2 (0.9)	69 (-)	
Monthly household income	B40 (<RM 4850)	143 (62.2)	75 (10.00)	
	M40 (RM 4851-10,971)	66 (28.7)	77 (9.25)	0.840
	T20 (>RM 10,971)	21 (9.1)	75 (12.00)	
Marital status	Single	229 (99.6)	76 (10.00)	0.145
	Married	1 (0.4)	-	
Educational level	Primary education	3 (1.3)	76 (-)	
	Secondary education	42 (18.3)	75.5 (16.50)	
	Degree	182 (79.1)	76 (9.25)	0.042^*^
	PhD or master’s degree	3 (1.3)	45 (-)	

* Kruskal–Wallis test, P<0.05

## Discussion

### Level of knowledge regarding unsafe abortion among young adults

The majority of the respondents were aware of various aspects that contribute to unsafe abortion. These aspects included abortions that are performed (1) in an environment that does not conform to minimal medical standards, (2) by untrained and unskilled personnel through dangerous methods such as inserting foreign bodies or using traditional concoctions, (3) by ingesting caustic substances and harmful chemical substances and (4) by inserting foreign objects into the vagina and uterus. These findings align with previous reports, showing that unsafe abortion is often conducted by individuals lacking the necessary skills, occurs in an environment that fails to conform to minimal medical standards and involves ingesting caustic substances, swallowing harmful chemical substances and inserting foreign objects/substances through the vagina or uterus.^17^ Additionally, most respondents recognised complications of unsafe abortion, such as death, haemorrhage, subfertility, uterine infections and negative mental or psychological impacts. Similarly, a previous study reported that the majority of female students in Ethiopia were aware of at least one type of complication from unsafe abortion.^19^

Apart from knowing the impacts and methods of unsafe abortion, most respondents were aware that abortion is allowed in several circumstances. The analyses revealed that many young adults agreed that abortion is not legally permitted in Malaysia, except when the pregnancy threatens the woman’s life or if the foetus might be born deformed. However, most respondents disagreed that abortion can be performed in instances where the family cannot support the child and if the woman is unmarried or wishes to further her studies. The respondents further agreed that women with unintended or unwanted pregnancies and no access to safe abortion are at risk of performing unsafe abortion. Similar findings were reported by previous research, whereby 47.7% of undergraduates in Sri Lanka agreed that abortion should be allowed to save a mother’s life.^16^ Another study demonstrated that the majority of health sciences students in Jordan disagreed with the idea of abortion, except if the pregnancy is threatening the mother’s life or caused by rape.^14^

In Malaysia, Section 312 Penal Code (1971) of the Attorney General’s Chamber postulates that abortion is permitted to be performed by medical practitioners registered under the Medical Act 1971 to save the pregnant woman’s life and preserve her physical and mental health. According to the law, individuals who voluntarily conduct an abortion without adhering to the set guidelines may face imprisonment of up to 3 years, fine or both.^20^ Furthermore, Section 315 Penal Code (1971) prohibits the act of abortion for women whose pregnancies are caused by rape or potentially deformed foetuses. However, possible exemption may persist if these pregnancy cases may inflict harm to the physical and mental health of the pregnant woman.^21^

According to the Malaysian National Fatwa Council (1990), abortion is allowed if the foetus is less than 120 days old, has severe disability and causes harm to the pregnant woman.^22^ The disability of the foetus must be verified by licensed medical practitioners before the pregnancy can be terminated. In this study, most respondents were aware of the law regarding abortion, with many opposing abortion if it is performed due to the failure to support the child and if the woman is not married or wants to further her studies. In contrast, most of them agreed that abortion may be considered if the child might be born deformed and if the pregnancy resulted from rape, especially if it would inflict negative impacts on the woman’s health.

### Level of attitudes towards unsafe abortion among young adults

The majority of the respondents disagreed with the idea of abortion when pregnant and agreed that unsafe abortion is a problem and a common situation among students. A previous study also reported similar findings, with participants agreeing that unsafe abortion is a problem (80.3%) and common among students (87.0%).^18^ Furthermore, the respondents in the present study had slightly different opinions regarding the abortion law in Malaysia, with 38.7% disagreeing and 35.2% agreeing that abortion is lawful in this country. Past research reported that the legalisation of abortion in Ethiopia was opposed by 55.6% of respondents, particularly due to the low awareness regarding the legalisation of induced abortion among the public.^19^ This suggests that the legal status of abortion is indeed a determinant for access to safe abortion services. Additionally, the respondents in the present study disagreed that abortion should be an option for unmarried pregnant adolescents. This mirrors previous findings whereby 52.2% of medical and health sciences students in Jordan opposed the idea of making abortion available to unmarried adolescents.^15^

Furthermore, most respondents agreed that legal restrictions often push women towards unsafe abortion and that making abortion services available in government healthcare facilities may increase the level of inappropriate sexual behaviours among the public. These findings align with a previous report,^16^ where respondents agreed that legal restrictions drive women to seek unsafe abortion (68.5%) and that offering abortion services through government health facilities increases sexual behaviours among women (63.7%). According to a previous study, women who are under pressure due to restrictive abortion laws are more prone to seeking abortion services elsewhere compared with the hospital setup.^18^ The availability of abortion services through government health facilities may have contributed to an increase in inappropriate sexual behaviours because women are aware of the option to terminate pregnancies without fully considering the short- and long-term complications associated with unsafe abortions. Consequently, once a pregnancy is resolved, there is a higher risk of women engaging in further inappropriate sexual activities.

Many respondents also agreed that the abortion decision should be made only by pregnant women. Past research showed that female students in Southwest Ethiopia agreed that the decision to have an abortion should rest solely with the pregnant woman, without involving her family or clinician.^19^ However, most respondents in the present study disagreed with the idea of considering abortion for foetuses with Down syndrome. Nevertheless, research has shown that most women have doubts about aborting their pregnancy despite knowing that the foetus has Down syndrome during prenatal screening. This may be influenced by physicians who are not well trained to provide the best counsel to pregnant women. It therefore serves as one of the factors that influence women’s decisions to keep their deformed pregnancy.^23^

In this study, the respondents agreed that committing an abortion is a sin and that women seeking abortions are often viewed as promiscuous. This finding aligns with previous research, whereby adolescents in the People’s Democratic Republic of Lao strongly agreed that abortion is sinful and that many people perceive women seeking abortions as a sign of promiscuity.^13^ The respondents also agreed that women should have access to safe abortion services to prevent them from resorting to harmful abortions performed in unsafe conditions or by untrained personnel. This is supported by a previous study, whereby respondents agreed that women should have access to safe abortion services, with women recording a higher level of agreement (71.0%) than men (65.0%), and that abortion can result in fatality (62.1%) if it is performed in unsafe situations.^13^

### Association of sociodemographic characteristics with the level of knowledge towards unsafe abortion

This study demonstrated an association of age and level of education with the level of knowledge regarding unsafe abortion. The respondents aged 23-24 years recorded a higher median score than the other age groups in terms of the level of knowledge. Past research also reported a similar association between the age range of 20-24 years and the level of knowledge,^18^ with young adults aged 21 years and above being five times more likely to possess knowledge about abortion than those below 20 years old.^24^ This suggests that young adults within this age range have good awareness regarding unsafe abortion, which can be attributed to their wider exposure to related topics and education during their studies.

An association between the level of knowledge and the level of education was also observed in this study, with the respondents with a bachelor’s degree recording a higher mean score than their counterparts. A study conducted in Indonesia reported that young adults with a good level of knowledge were primarily those in bachelor’s degree programmes (66.7%) compared with those in senior high school (46.5%).^25^ This may be attributed to the fact that young adults in bachelor’s degree programmes typically have a higher level of knowledge than those in senior high school due to factors such as more in-depth education, greater maturity, better access to academic resources and exposure to specialised courses. These elements help to enhance their understanding of complex issues such as abortion, while senior high school students may not yet have the opportunity to engage with these topics in greater detail.

Conversely, the present study demonstrated no association of marital status, monthly family income and sex with the level of knowledge regarding unsafe abortion among young adults in Kuantan, Pahang. This contradicts previous reports, showing that marital status has a significant association with the level of knowledge regarding abortion. Moreover, students who are not in a relationship, in a relationship and married are reported to be more knowledgeable than those who have never been in any relationship. This is because students with relationship histories may have information regarding sexual intercourse and can therefore avoid unwanted pregnancy.^24^ Previous studies also reported a significant association between family income and knowledge of induced abortion among female students.^19,24^ Respondents with no financial problem had more access to information regarding sexual intercourse and abortion, especially through social media. Another study reported that sex was associated with the level of knowledge regarding unsafe abortion, with female individuals having better knowledge than their male counterparts.^16^

*Association of sociodemographic characteristics with the level of attitudes towards unsafe abortion* This study revealed an association of religion and level of education with the level of attitudes towards unsafe abortion among young adults in Kuantan, Pahang. The highest median score was obtained by Buddhists, followed by Muslims and Hindus. A similar finding was reported by a past study involving female students in private colleges in Ambo Town, Oromia Regional State, Ethiopia, whereby Protestant participants were 0.48 times less likely to exhibit a positive attitude than those from the Orthodox religion.^24^ Differences in attitudes related to religion may have been shaped by variations in religious teachings, cultural practices, community influences and access to reproductive health information. Past research also suggested that religious beliefs might influence students’ attitudes towards abortion.^15^

Additionally, the respondents with a degree-level education recorded a higher median score than the other educational groups. A previous study reported that attitudes towards abortion were significantly influenced by the year of study. First-year university students were more likely to view abortion as murder and expressed more negative opinions than upper-level students. They were also less likely to support abortion in cases involving foetal congenital abnormalities.^15^ Another study also reported that individuals who graduated from high school demonstrated 4.9 times more correct knowledge and 17.5 times more correct practices than those who did not attend high school. It is common for students with higher levels of education to be more aware of reproductive issues and actively seek information on this topic.^26^

### Limitations

One limitation of this study is the questionnaire’s availability in the English language only. Although the target group was expected to have basic English skills, using a non-native language might have affected their understanding. However, the respondents were allowed to ask for clarification during data collection. Additionally, the use of English might have introduced sampling bias and affected the generalisability of the findings across all Malaysian youths, especially those who are more comfortable with the Malay language.

Furthermore, the questionnaire used in this study was adapted from five different instruments, with only one having documented validity and reliability testing. The other four questionnaires were reported to undergo pilot testing, with adjustments made to improve clarity. The questionnaire items were selected based on their relevance to the research objectives, but no formal content validation, face validation or reliability testing was conducted prior to distribution. This could be a limitation that might affect the accuracy and generalisability of the findings. Therefore, a full validity and reliability assessment should be performed in future research.

Another notable limitation of this study is the imbalance in several sociodemographic variables, particularly in the overrepresentation of Malays (97.0%), female respondents (89.6%) and students (88.3%). These distributions largely reflected the actual demographic characteristics of the population studied, which was aged 15–24 years, which is consistent with the national and regional population statistics for Kuantan, Pahang, Malaysia. The predominance of students also aligns with the national enrolment patterns among individuals aged 15–24 years, where a large proportion is enrolled in secondary or tertiary education. This is also reflected in our sociodemographic data, whereby 79.1% and 18.3% of the respondents were pursuing a degree and enrolled in secondary school, respectively. Additionally, the questionnaire distribution method might have unintentionally resulted in a female-dominant sample. This can be attributed to several factors, such as women being more responsive to health-related surveys, having greater interest or concern regarding reproductive health topics and being more willing to participate in voluntary research, especially when shared through peers and online. Such skewed representation limits the generalisability of the findings to a broader Malaysian youth population, especially concerning other ethnicities, sex and non-students. The present study was also designed as an initial exploratory research, aiming to describe the levels of knowledge and attitudes among the target population rather than to establish causal or predictive associations. Consequently, the use of univariate analysis limits the ability to infer causal relationships. Future research should employ multivariate analysis to further explore the associations and confounding factors with a larger and more diverse sample.

## Conclusion

Most respondents demonstrated a clear understanding of unsafe abortion methods, their complications and the legal context surrounding abortion in Malaysia. The respondents were aware of the risks associated with unsafe abortion and acknowledged that it is legally permissible only in specific circumstances, such as to save the mother’s life or in cases of foetal deformity or rape. However, the respondents’ attitudes towards abortion varied, with many disagreeing with abortion in certain situations, such as for unmarried women or if a family cannot support the child. Age and educational level were positively correlated with knowledge. Additionally, religious beliefs and educational level also influenced the attitudes towards unsafe abortion. Therefore, this study emphasises the need for comprehensive reproductive health education and accessible services to prevent unsafe abortions, particularly among young adults. It also calls for policies that address socioeconomic and geographic disparities to ensure all individuals have the knowledge and support they need to make informed reproductive health decisions.

## Data sharing statement

The data supporting the findings of this study can be obtained upon request from the corresponding author. However, they are not publicly accessible, as they contain information that could compromise the privacy of the research participants.

**How does this paper make a difference in general practice?**

- This study emphasises the importance of raising knowledge and attitudes towards unsafe abortion risks, methods and complications, to improve public health knowledge.
- The findings demonstrate the need for clearer public health policies and enhanced access to safe abortion services to reduce unsafe practice and promote safer reproductive health.
- The findings emphasise the need for tailored interventions that address age, education and religious belief disparities, ensuring equitable access to reproductive health information and services.
- This research highlights the role of culture and religion in shaping public views on abortion and the need to reduce stigma and encouraging open dialogue about reproductive health.
